# Histone acetyltransferase CBP-related H3K23 acetylation contributes to courtship learning in *Drosophila*

**DOI:** 10.1186/s12861-018-0179-z

**Published:** 2018-11-20

**Authors:** Kai-Le Li, Lei Zhang, Xiao-Mei Yang, Qiang Fang, Xue-Fang Yin, Hui-Min Wei, Ting Zhou, Ya-Bin Li, Xue-Lin Chen, Fan Tang, Yong-Hao Li, Jian-Feng Chang, Wei Li, Feng Sun

**Affiliations:** 1 Research Center for Translational Medicine at East Hospital, School of Life Sciences and Technology, Tongji University, Shanghai, 200092 China; 2 Alpha Institute of Natural Medicine, Tongji University, Shanghai, 200120 China; 3 Tsingdao Advanced Research Institute, Tongji University, Qingdao, 266071 China; 4 Tongji Hospital, Tongji University School of Medicine, Shanghai, 200065 China

**Keywords:** H3K23ac, *dCBP*, Courtship learning, *Drosophila melanogaster*

## Abstract

**Background:**

Histone modifications are critical in regulating neuronal processes. However, the impacts of individual histone modifications on learning and memory are elusive. Here, we investigated the contributions of histone H3 lysine modifications to learning and memory in *Drosophila* by using histone lysine-to-alanine mutants.

**Results:**

Behavioural analysis indicated that compared to the H3WT group, mutants overexpressing H3K23A displayed impaired courtship learning. Chromatin immunoprecipitation analysis of H3K23A mutants showed that H3K23 acetylation (H3K23ac) levels were decreased on learning-related genes. Knockdown of CREB-binding protein (CBP) decreased H3K23ac levels, attenuated the expression of learning-related genes, led to a courtship learning defect and altered development of the mushroom bodies. A decline in courtship learning ability was observed in both larvae and adult treatments with ICG-001. Furthermore, treatment of *Drosophila* overexpressing mutated H3K23A with a CBP inhibitor did not aggravate the learning defect.

**Conclusions:**

H3K23ac, catalysed by the acetyltransferases *dCBP*, contributes to *Drosophila* learning, likely by controlling the expression of specific genes. This is a novel epigenetic regulatory mechanism underlying neuronal behaviours.

**Electronic supplementary material:**

The online version of this article (10.1186/s12861-018-0179-z) contains supplementary material, which is available to authorized users.

## Background

Learning and memory can be broadly defined as lasting alterations to behavioural outputs that are produced in response to a transient environmental input [1]. Understanding the cellular and molecular mechanisms that underlie these activities is one of the central goals of the neuroscience community [1]. Although the learning field has been a focus of research in past decades, the molecular and epigenetic mechanisms that underlie learning and memory are still not well understood. Recently, studies have suggested that histone modifications regulate synaptic plasticity, memory formation and intellectual disability disorders, likely by controlling gene transcription [2–4]. However, dissecting the precise functional roles of individual histone modifications has been challenging.

Histone modifications are chemical groups, such as acetyl and methyl groups, that are enzymatically added to and removed from particular amino acids in the histones *H2A*, *H2B*, *H3* and *H4*, which make up the octamer around which the DNA duplex is wound. Histone acetylation, which usually affects the interaction between DNA and histones and further regulates gene transcription [5–8], is catalysed by histone acetyl transferases (HATs), whereas histone deacetylases (HDACs) are responsible for the removal of acetyl groups [9, 10]. HATs are mainly classified into three basic subfamilies: the Gcn5-related N-acetyltransferases (GNATs), MYST (*MOZ*, *Ybf2*/*Sas3*, *Sas2* and *Tip60*) and *p300/CBP* [11]. With histone methylation, up to three methyl groups can be added to lysine residues, leading to mono-, di-, or trimethylation patterns that affect the transcriptional regulation of locally wound DNA[12].

According to recent genetic and pharmacological evidence, changes in the activity of histone-modifying enzymes impact cognition [13]. For instance, histone lysine acetylation is tightly involved in the control of learning and memory [14]. Several HATs modulate neuronal activity, synaptic plasticity and memory formation [14]. In *Aplysia*, when a sensory neuron is stimulated by 5-hydroxytryptamine (5-HT), *CBP* is recruited to the C/EBP (CAAT box enhancer binding protein) promoter, followed by the acetylation of specific lysine residues on different histones, which further induce gene expression [15]. Moreover, mice with a partial or complete loss of *CBP* function exhibit reduced histone acetylation levels and impairments in long-term memory ability [16–19]. HDACs negatively modulate memory formation [20, 21]. Mice that overexpress *HDAC2* exhibit impaired memory formation, whereas *HDAC2* knockout mice exhibit enhanced memory formation [3]. In *Drosophila*, *HDAC4* overexpression in adult mushroom bodies impairs long-term courtship memory [22]. Based on the role of HDACs in learning and memory, the administration of HDAC-specific small-molecule inhibitors, such as sodium butyrate (NaB), valproate, or trichostatin A (TSA), significantly improves cognitive deficiency [23]. Thus, HDAC inhibitors can potentially treat cognitive disorders [24]. In addition, histone methylation and phosphorylation participate in learning and memory. Histone H3S10 phosphorylation and H3K36 trimethylation are increased in mice with improved memory after treatment with an inhibitor of nuclear protein phosphatase 1 (*PP1*) [25]. *EZH2*, the major methyltransferase of histone H3K27, regulates adult hippocampal neurogenesis. *EZH2* knockout mice have impaired spatial learning and memory, contextual fear memory, and pattern separation [26].

Furthermore, correlational evidence indicates that histone post-translational modifications, predominantly histone acetylation, are modulated by experiences, such as learning and memory [13]. Early studies using radioactive acetate incorporation indicated that histone acetylation increases in the hippocampus after training compared with that in untrained controls, whereas histone acetylation decreases in other brain regions, such as the cortex [27]. Various residues on histone tails can be acetylated in response to training [14, 28]. In 32/36 studies in which general changes in HAT or HDAC were investigated, improved learning was positively correlated with a global increase in acetylation, regardless of the learning task or species investigated [12]. Considering the evidence, histone modifications likely play a key role in learning and memory.

Histone-modifying enzymes, such as HATs and HDACs, have multiple functions and substrates, and genetic evidence alone may be insufficient to definitively demonstrate their involvement in a specific transcriptional process [13]. As there is currently no means to selectively prevent the hyperacetylation of histones while preserving the hyperacetylation of non-histone substrates, direct evidence that demonstrates a causative role for histone acetylation in mediating the effects of HDAC remains lacking [13]. Clear discrimination between cause and effect is required to understand the roles of individual histone modifications in neuronal plasticity, learning, memory, and neuropsychiatric disorders. Previous reports had indicated that histone lysine mutants are powerful tools to study the functions of histone-specific lysine modifications [29, 30]. To determine the causative role of individual histone modifications in learning and memory, we constructed histone lysine-to-alanine mutant *Drosophila* strains, characterized the effects of each mutant on learning and memory, and identified an association between H3K23 acetylation and courtship learning in *Drosophila*. Furthermore, we observed that a loss of *dCBP*, a histone *H3* lysine 23 acetyltransferase, also impaired neuronal gene activation and resulted in defects in courtship learning. However, inhibition of *dCBP* in the H3K23A overexpression line did not have additive effects on learning. Therefore, *dCBP*-related H3K23 acetylation is required for successful learning.

## Results

### Histone H3K23A mutation affects courtship learning in *Drosophila*

**Fig. 1:**
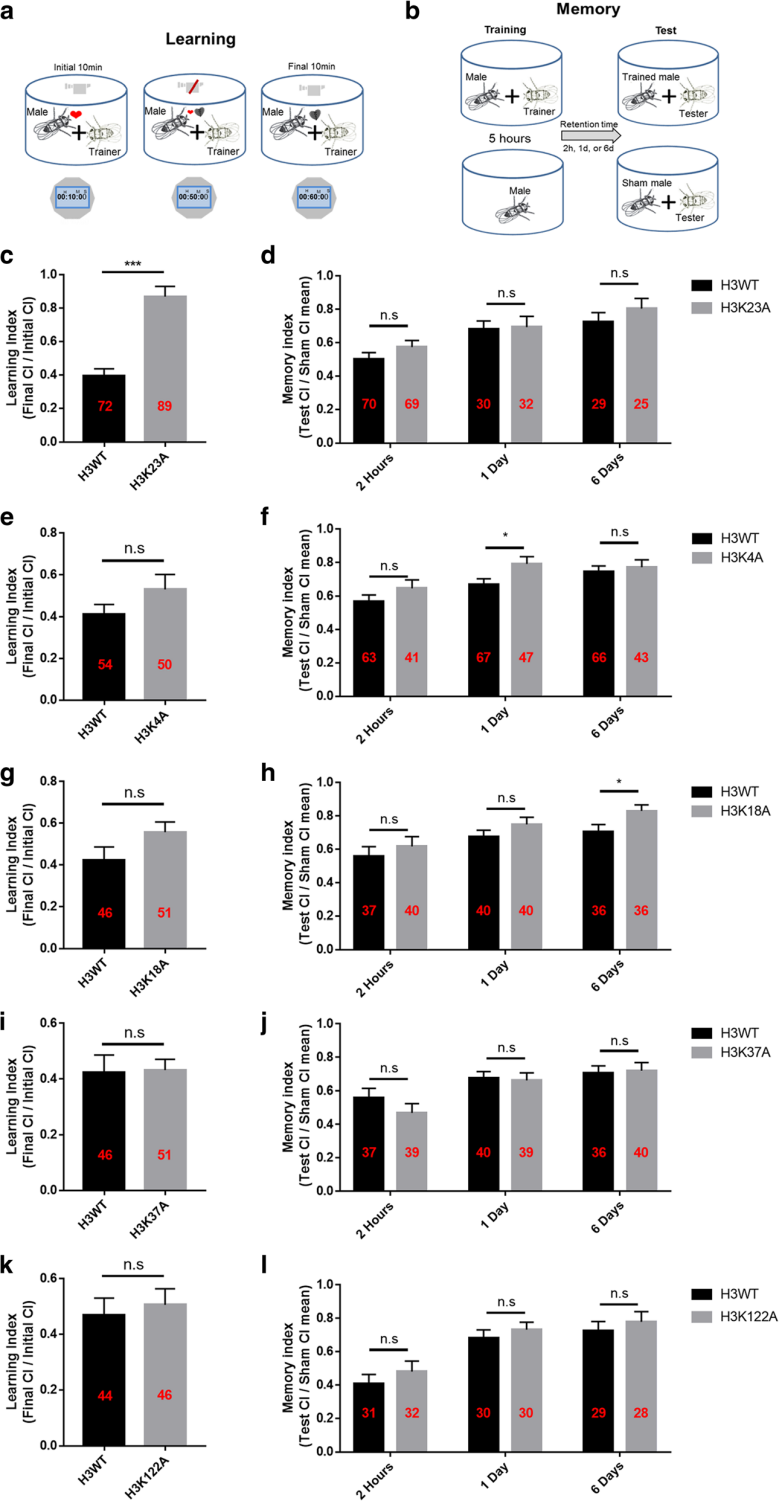
Histone H3K23A mutation affects *Drosophila* courtship learning. **a**, **b** Schematic presentation of *Drosophila* courtship learning (**a**) and memory (**b**). For learning, each male was paired with a mated female (trainer) for 1 hour. The learning index of courtship was the time spent during the final 10 min vs. the initial 10 min. For memory, each male was allowed to train for 5 hours. The memory index was calculated as the ratio of the courtship level of each trained male to the mean of sham males. An LI or MI score ≥1 indicates no learning or memory. (CI) Courtship learning and memory analysis of H3K23A, H3K4A, H3K18A, H3K37A and H3K122A overexpression flies. Histone mutant are driven by Ok107-GAL4 Courtship learning indexes (**c**, **e**, **g**, **i**, **k**) or memory indexes (**d**, **f**, **h**, **j**, **l**) were measured in various of mutant H3 overexpression flies. H3K23A overexpression flies exhibited significant difference in learning (**c**), and overexpressing H3K4A or H3K18A showed memory defect (**f**, **h**). Unpaired t-test was used for statistics. Error bars represent the standard error of the mean; the number of samples was indicated in the bar. n.s., not significant. *p<0.05, **p<0.01, ***p<0.001

To identify the causative roles of individual histone modifications in learning and memory, various *Drosophila* strains with UAS-histone H3 lysine-to-alanine mutations were constructed. These mutant strains overexpressed histone H3 with specific lysine residues changed to alanine residues. First, to validate whether the overexpressed histone H3 mutants, which were fused with GFP, were incorporated into the chromatin, we examined the expression of GFP on polytene chromosomes from third-instar larvae of the histone mutant lines via immunofluorescence assays. We found that GFP was indeed expressed in the polytene chromosomes (Additional file 1), suggesting that these histone mutants were also incorporated into the chromatin. Next, we performed a courtship-suppressing experiment with an OK107-GAL4 driver, which drives histone mutation expression specifically in the mushroom body (the key tissue for learning and memory in *Drosophila*). Unsuccessful courtship reduces the subsequent courtship behaviour of male flies.[31] This experiment is one of the major paradigms used to study learning and memory in *Drosophila* (Fig. 1a-b). According to the calculation method, learning and memory levels are inversely related to the courtship indexes. We therefore tested the learning and memory levels of the strains overexpressing histone H3 mutants. Overexpression of H3K23A significantly impaired the courtship learning of *Drosophila* after training for 1 hour compared with that observed in the H3WT group (control) (Fig. 1c, Additional file 9, videos 1-2). This result indicated that the modification of the H3K23 residues may influence learning in *Drosophila*. In contrast, we did not observe significant changes in learning levels in *Drosophila* carrying other H3 mutants such as H3K4A, H3K18A, H3K37A and H3K122A (Fig. 1. e, g, i, k).

Then we asked whether the memory levels changed in these *Drosophila* lines. Considering the lower learning abilities of H3K23A group, we decided to extend the training time from 1 hour to 5 hours. We used another courtship learning experiment to value the learning level of H3K23A overexpression flies after a 5-hours-training. No significant difference of learning index was observed between the groups of overexpressing H3WT and H3K23A after training for 5 hours (Additional file 2, p=0.1379). Thus, we used this training method to study the memory levels of *Drosophila* in this article.

No obvious effect on memory was observed at 2 hours, 1 day and 6 days in the H3K23A, H3K37A, and H3K122A overexpression flies after training for 5 hours (Fig. 1d). To our surprise, some minor changes in memory ability were observed in the H3K4A group (1day, p=0.0297) and H3K18A group (6 days, p=0.0294) (Fig. 1f, h). Considering the slight but not significant changes in the learning index (Fig. 1e, g), modifications to H3K4 and H3K18 may slightly affect learning and memory in *Drosophila*.

In addition, no significant differences in the total courtship time were observed except for the group overexpressing the H3K37A mutant. (Additional file 3a, b, c, d and e). To determine whether the initial courtship time (initial CI) affected the learning index, we analysed the relationship between the initial CI and the learning index in these *Drosophila* lines. We combined all H3WT groups and bounded them by the median. No significant difference in the learning index was found between the two subgroups (Additional file 3f). The same analysis was performed with the other groups, and no significant changes were observed, except in the H3K18A mutant (Additional file 3g, h, i and k). It appeared that there was no significant relationship between the initial courtship time and the learning index.

According to the data, even though the H3K4A and H3K18A lines showed similar trends in learning and memory, these phenotypes were weak. As the H3K23A overexpression line showed a large difference in learning but not memory, it was very interesting to study how H3K23A regulates the learning behaviour. Therefore, we focused on H3K23, which might be a major site of modification to regulate learning.

### Decreased H3K23ac impairs neuronal gene activation in the H3K23A overexpression line

**Fig. 2:**
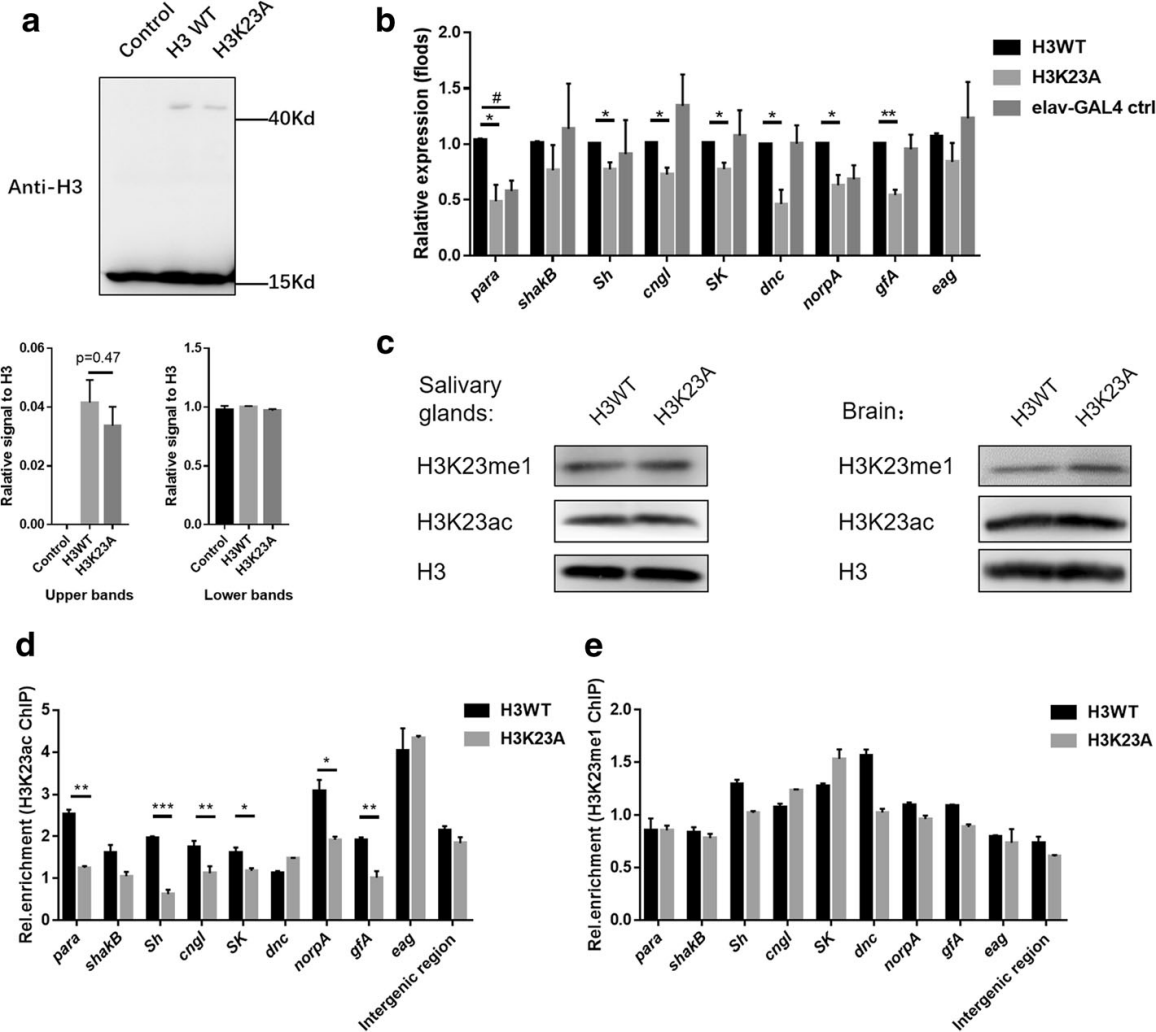
Decreased H3K23ac levels impair neuronal gene activation in H3K23A mutants. **a** A western blotting was used to calculate the ratio of exogenous *H3*. The data was measured by the software Image J. N=3 biological replicates. **b** The expression levels of neuronal genes were examined by RT-qPCR in the larval brains. The mRNA levels of neuronal genes were normalized to the levels of rp49. Unpaired t test with Welch’s correction was used. N=3 biological replicates. **c** The H3K23ac and the H3K23me1 levels are examined by western blotting. The samples were collected from salivary gland and larval brains, respectively. **d**-**e** The H3K23A mutation reduces the occupancy of the H3K23ac marker in the neuronal genes. The enrichment of H3K23ac and H3K23me1 were normalized to input. The intergenic region was used as the euchromatic control region. Unpaired t-test was used. Error bars represent standard error of the mean (N=2 biological replicates). *p<0.05, **p<0.01, ***p<0.001, #*p*<0.05 (elav-GAL4 group compared to H3WT group)

Before investigating how H3K23A affects learning, we used a western blot experiment to test the ratio of exogenous H3. Exogenous H3 fused with GFP showed a 42-KDa band, which ran higher than the endogenous H3 band of approximately 15-KDa (Fig. 2a). At the same time, we noticed that the overexpression of both H3WT-GFP and H3K23A-GFP accounted for approximately 4 percent of the total H3 protein (Fig. 2a).

As courtship learning was impaired in the H3K23A overexpression line, we next asked whether the expression of neuronal genes related to *Drosophila* learning were also affected in this line. To test this, an RT-qPCR assay was conducted to determine the expression levels of neuronal genes, including voltage-gated ion channels (*Sh*, *eag* and *para*), ligand-gated channels (*SK*, *Cngl* and *gfA*), phospholipase C (*norpA*), and phosphodiesterase (*dnc*) [32–40]. The expression of these candidate neuronal genes, except for *eag* and *shakB*, significantly decreased in the H3K23A overexpression line. (Fig. 2b). Four housekeeping genes were tested as the additional controls at the same time. Compared to the H3WT groups, all these four genes in the H3K23A overexpression groups showed no significant differences (Additional file 4a). At the same time, compared to the H3WT groups, there were only a little change in the elav-GAL4 control groups (Fig. 2b, Additional file 4a).

*Cngl* and *gfA* can regulate the activity of calcium signalling pathways [41, 42], and calcium signalling pathways have emerged as key players in learning and memory[43–45]. Therefore, we used calcium imaging to monitor the Ca^2+^ responses induced by KCl stimulation of larval brains. Fura-red was used as a red Ca^2+^ indicator dye combined with GFP-tagged brain. Ca^2+^ signals can be presented as values (ΔR/R_0_) of the relative change rate in fluorescence intensity (ΔR) normalized to the baseline fluorescence rate (R_0_). The H3K23A mutant was driven by Elav-GAL4. H3K23A mutant larval brains showed a lower calcium response after KCl stimulation than the control group (Additional file 4b). This result demonstrates that calcium signalling pathways were impaired in the brains of the H3K23A overexpression line.

A previous study reported that acetylation occurs in *Drosophila* at the H3K23 residue [46], although methylation occurs at the H3 lysine 23 site in *Tetrahymena* and *C. elegans* [47]. H3K23ac activates the transcription of genes. However, H3K23 methylation (H3K23me), including H3K23me1, H3K23me2 and H3K23me3, is associated with heterochromatin. Therefore, we examined global H3K23ac and H3K23me1 levels followed the overexpression of H3WT or H3K23A. Both of H3K23ac and H3K23me1 showed no difference in either salivary glands or the brain. (Fig. 2c).

We hypothesized that these neuronal genes were regulated in a gene-specific manner. To test this, we performed a chromatin immunoprecipitation (ChIP) assay with larval brains using antibodies against H3K23ac or H3K23me1 followed by real-time PCR. The PCR primers were designed to amplify the sequences of the coding regions of the affected neuronal genes. The intergenic regions were used as controls[46]. Compared with that in H3WT group, the enrichment of H3K23ac was significantly decreased among most of these neuronal genes in the group overexpressing the H3K23A mutant (Fig. 2d), indicating that H3K23ac is involved in the regulation of gene expression. However, there were no significant differences in H3K23me1 enrichment levels in these neuronal genes (Fig. 2e). Because we lacked the ChIP-grade antibodies against H3K23me2 or H3K23me3, we could not exclude whether the possibility that these two modifications contribute to the regulation of neuronal genes expression. In summary, H3K23ac, instead of H3K23me1, seemed to play a role in regulating learning in *Drosophila* by activating the expression of neuronal genes.

### Knockdown of *dCBP* expression in the *Drosophila* nervous system decreased H3K23ac levels and led to a defect in courtship learning ability

**Fig. 3:**
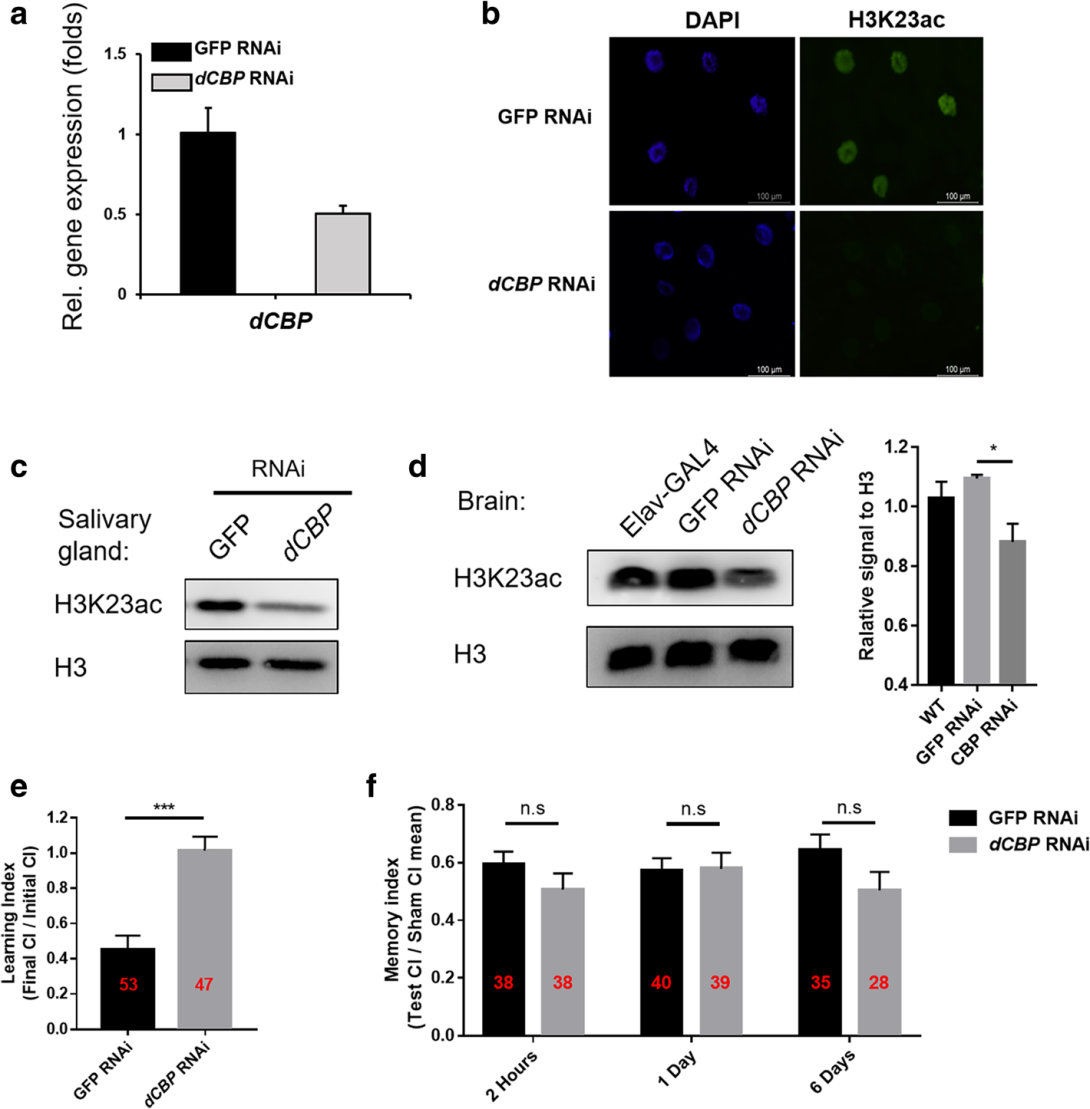
Knockdown of *dCBP* expression in the *Drosophila* nervous system decreased H3K23ac levels and led to a defect in courtship learning ability. **a** The RNAi efficiencies of CBP in larval brain-discs was detected by RT-qPCR. Female UAS-GFP RNAi (control line), UAS-*dCBP* RNAi flies were crossed with Elav-GAL4 males. *rp49* was used as the reference gene. Error bars represent standard error of the mean. **b** Salivary gland immunostaining was performed in *dCBP* RNAi flies with anti-H3K23ac. RNAi induced knockdown was driven by SG-GAL4. **c** Western blotting analysis of H3K23ac protein levels from whole-salivary gland lysates after the depletion of *dCBP.* **d** Western blot for H3K23ac revealed decreased H3K23ac expression in the brain lysates of *dCBP* RNAi flies. The quantified data was measured by the software Image J. N=3 biological replicates. **e** Courtship learning analyses of *dCBP* RNAi flies. Compared to GFP RNAi flies, the *dCBP*-knockdown flies exhibited learning defects. **f** *dCBP* RNAi exhibited normal courtship memory. *dCBP* knockdown in the mushroom body of adult flies was driven by Ok107-GAL4. Unpaired t-test was used for statistics. Error bars represent the standard error of the mean; the number of samples was indicated in the bar. n.s., not significant. ***p<0.001

To further study the functions of H3K23ac post-translational modification in learning, we determined which histone acetyltransferase acetylates H3K23 in the nervous system in *Drosophila*. GCN5 can acetylate the H3K23 site in yeast [48] and is the major acyltransferase for two distinct histone residues, H3K9 and H3K14, in *Drosophila*[49], while *dCBP* catalyse H3K23 acetylation in flies [46, 50]. Thus, we suspected that *dCBP* is one of the enzymes related to H3K23ac, that regulates courtship learning. Therefore, we constructed transgenic RNAi flies with an Elav-GAL4 driver to disrupt the expression of *dCBP*. The RNAi efficiency was validated by RT-qPCR, and the mRNA level of *dCBP* was greatly reduced in larval brains with the pan-neuronal driver Elav-GAL4 (Fig. 3a). Then, we examined H3K23ac levels in polytene chromosomes from third-instar larvae of the *dCBP* RNAi lines via immunofluorescence assays. This RNAi line was driven by salivary gland-specific GAL4 (SG-GAL4). Indeed, the levels of H3K23ac were significantly decreased in the *dCBP* RNAi line (Fig. 3b), and this result was confirmed by western blot (Fig. 3c). These results suggested that *dCBP* can catalyse H3K23ac in *Drosophila*.

Furthermore, to determine whether *dCBP* is responsible for the acetylation of H3K23 in the nervous system, we constructed a neuron-specific *dCBP* knockdown fly strain with the pan-neuronal driver elav-GAL4. Consistently, *dCBP* depletion in the larval nervous system resulted in a striking reduction in H3K23ac levels (Fig. 3d). Thus, we concluded that *dCBP* is likely the histone acetyltransferase for H3K23 in the nervous system of *Drosophila*. Nevertheless, we could not exclude the possibility that additional epigenetic enzymes may contribute to modifications on the H3K23 site.

Next, we tested whether *dCBP* RNAi *Drosophila* exhibited a phenotype like that of the H3K23A overexpression line, which had exhibited defects in learning ability according to the courtship suppression behavioural analysis. The Ok107-GAL4 driver was used (the GFP RNAi line was used as a control). As expected, when tested in the behavioural assay, the *dCBP* RNAi line exhibited learning defects (Fig. 3e, Additional file 9, videos 3-4). As with overexpression of the H3K23A mutant, we did not observe obvious effects on memory (Fig. 3f). In summary, *dCBP* may contribute to courtship learning, likely by modulating H3K23 acetylation. Surprisingly, *dCBP* RNAi flies appeared to be more active at the initial time of courtship (Additional file 5:a). The initial courtship time did not affect the learning index of the GFP RNAi group but did affect that of the *dCBP* RNAi group. There seems to be some interaction between these two effects (Additional file 5:b-c). It indicating that *dCBP* RNAi might regulate other functions besides learning.

### Impaired learning ability in *dCBP* RNAi *Drosophila* may due to decreased neuronal gene expression, which is regulated by the level of H3K23ac

**Fig. 4:**
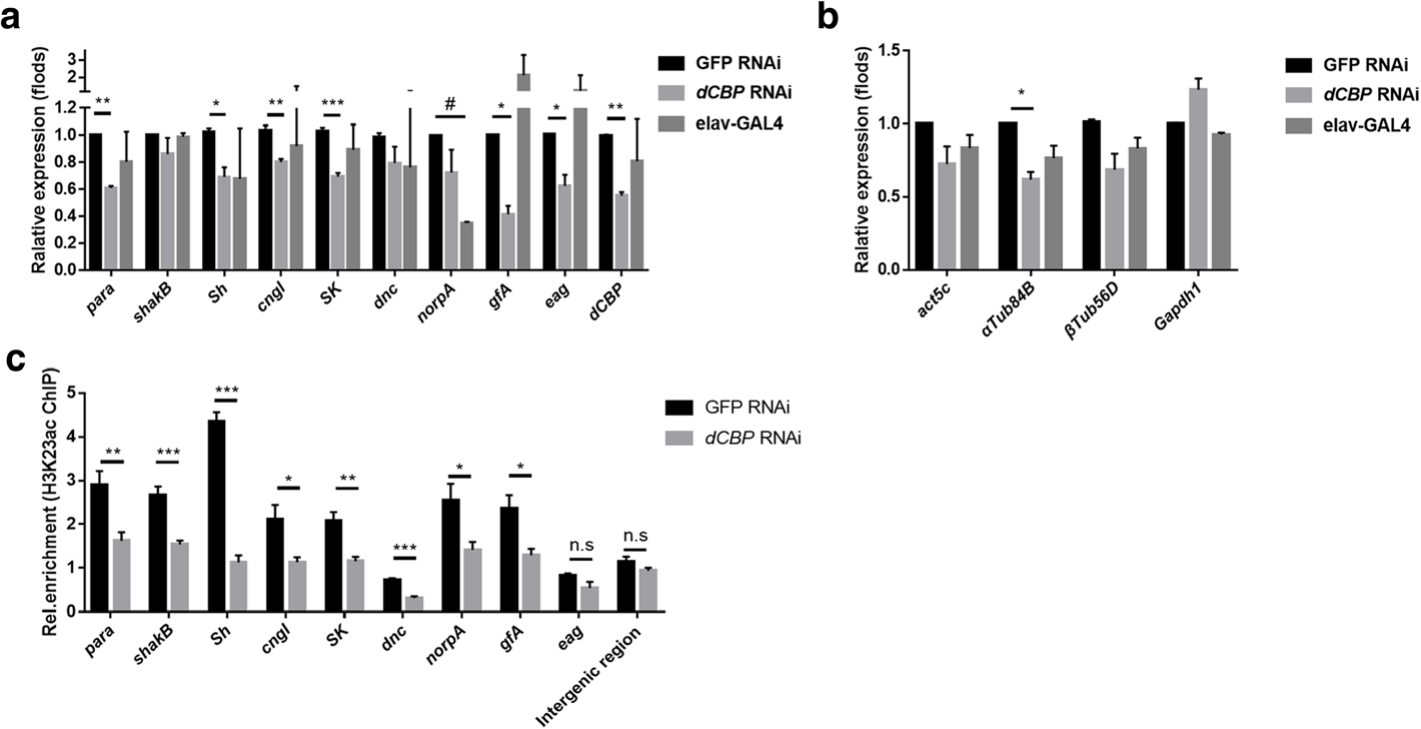
Impaired learning ability in *dCBP* RNAi *Drosophila* may due to decreased neuronal gene expression, which is regulated by the level of H3K23ac. **a**-**b** The expression levels of neuronal genes were examined by RT-qPCR in the larval brains of *dCBP* RNAi. The mRNA levels of neuronal genes were normalized to the levels of *rp49*. Unpaired t-test with Welch’s correction was used. N=3 biological replicates. **c** The ChIP assay and RT-qPCR analysis validated the decreased enrichment of H3K23ac in the neuronal genes after the silencing of *dCBP* in the larval brains. The enrichment of H3K23ac was normalized to input. The intergenic region was the euchromatic control region. Error bars represent the standard error of the mean. (N=2 biological replicates). n.s., not significant, *p<0.05, **p<0.01, ***p<0.001, #*p*<0.05 (elav-GAL4 group compared to H3WT group)

To examine whether *dCBP* RNAi also affected the expression of neuronal genes related to *Drosophila* learning, we conducted an RT-qPCR assay to determine the expression levels of neuronal genes. The expression of candidate neuronal genes was significantly decreased in *dCBP* RNAi flies, except for *shakB*, *dnc* and *norpA* (Fig. 4a). The four housekeep genes were examined at the same time, and we observed a down-regulation in the *beta-tubulin* but not in *gapdh1* (Fig.4b). Consistently, the expression of most neuronal genes we examined were decreased in both the *dCBP* RNAi and the H3K23A overexpression groups (Fig. 3b, Fig. 4a-b). It seems that *dCBP* and its target H3K23ac have similar functions in regulating neuronal gene expression. Thus, we hypothesized that the expression levels of these neuronal genes are related to H3K23ac levels, which is catalysed by *dCBP.* To test this hypothesis, we performed a ChIP assay with control (GFP RNAi) and *dCBP* RNAi larval brains using an antibody against H3K23ac followed by real time-PCR. Compared with those in the control group, H3K23ac enrichment levels were significantly decreased in most of these neuronal genes, except for *eag* (Fig. 4c), which confirmed with the global down-regulation of H3K23 level (Fig. 3c-d, Fig. 4c). Combined with the results of the previous ChIP assay, which revealed an obvious reduction in H3K23ac levels in the neuronal genes in larval brains overexpressing the H3K23A mutant (Fig. 2d), these data demonstrated that H3K23ac, catalysed by *dCBP*, contributes to courtship learning in *Drosophila* likely by regulating the expression of neuronal genes related to learning. To be noticed, the inconsistency in expression levels and enrichment levels in some genes such as *shakB* suggested that these genes might be regulated by other modifications beside H3K23ac.

### Inhibition of *dCBP* in the H3K23A overexpressing strain did not aggravate the learning defect

**Fig. 5:**
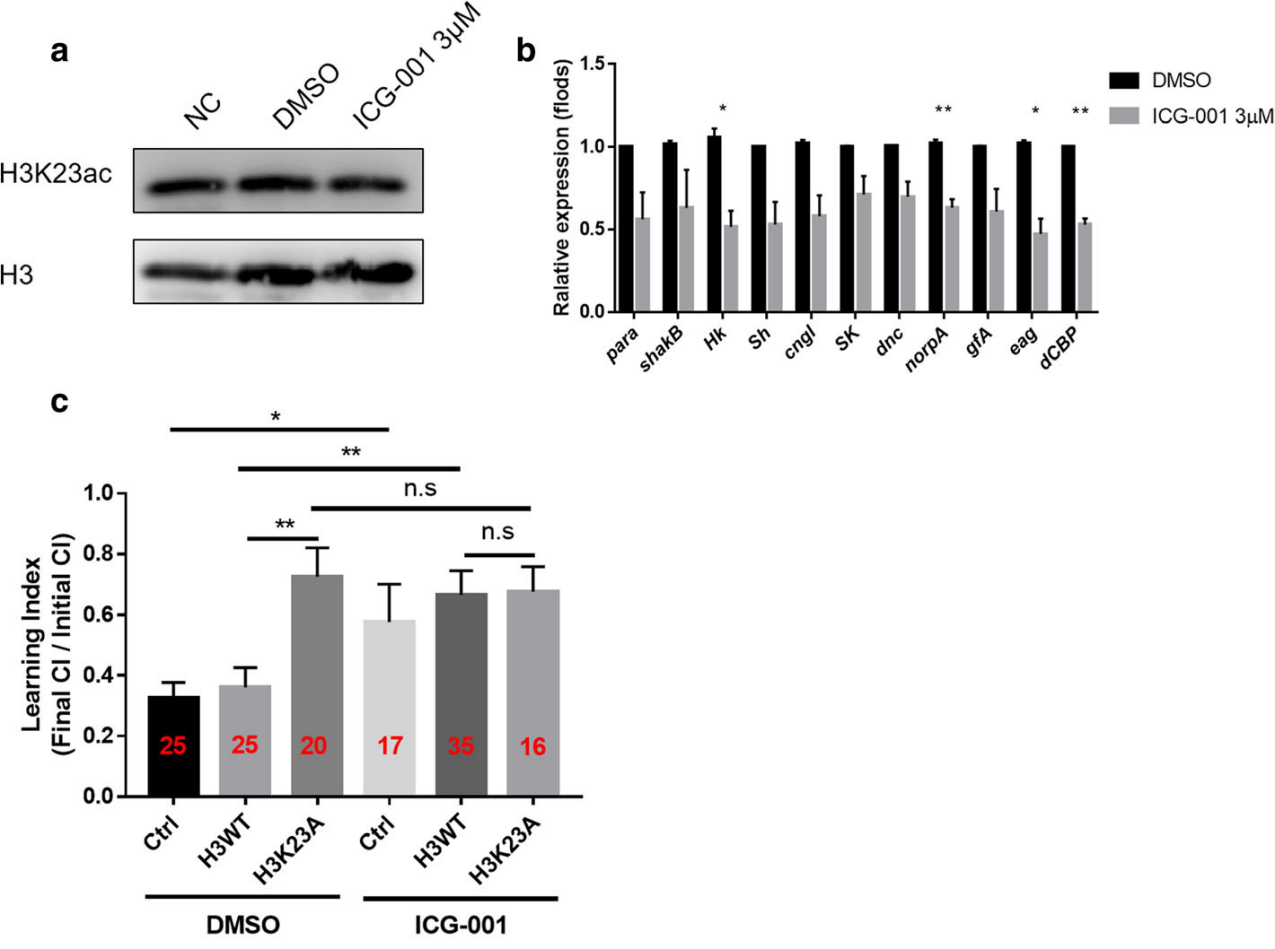
Inhibition of CBP in the H3K23A overexpressing strain did not aggravate the learning defect. 3μM ICG-001 or equal volume DMSO were mixed into the food. The flies were raised in the medium with either ICG-001 ow DMSO. **a** After treatment with ICG-001, the H3K23ac level in the larval brains were detected by western blotting. The data was measured by the software Image J. **b** The expression levels of neuronal genes were examined by RT-qPCR in the larval brains followed the treatment with ICG-001 in the elav-GAL4 strain. The mRNA levels of neuronal genes were normalized to the levels of *rp49*. Unpaired t-test with Welch’s correction was used. Error bars represent the standard error of the mean. *N*=3 biological replicates. **c** Courtship learning analyses of flies. Learning indexes were calculated after treatment with ICG-001. Unpaired t-test was used for statistics. Error bars represent the standard error of the mean; the number of samples was indicated in the bar. n.s., not significant. *p<0.05, **p<0.01

Based on the data above, we then asked whether *dCBP* is one of the major enzymes that regulates learning by catalysing H3K23ac. To investigate this question, we treated flies with ICG-001, an inhibitor specific to CBP[51]. First, a western blotting assay was used to assess the H3K23ac level after treatment with ICG-001. As expected, the H3K23ac level was slightly down-regulated in larval brains after treatment with ICG-001 (Fig. 5a). Then we tested the expression levels of neuronal genes in larval brains after treatment with ICG-001. Compared to those in the control groups (DMSO), the expression levels of these neuronal genes decreased after treatment with ICG-001 (Fig. 5b). Interestingly, when we treated flies with ICG-001, we observed the down-regulation of *dCBP* expression (Fig. 5b), which might have an additional effect on the regulation of gene expression.

Furthermore, a courtship suppression experiment was conducted to investigate whether the effect of *CBP* downregulation and H3K23A mutant overexpression are additive. In this experiment, we observed learning defects in both Elav-GAL4 control group and the H3WT overexpression group treated with ICG-001 (Fig. 5c, lanes 1, 2, 4 and 5). There were no additive effects in the H3K23A overexpression group which treated with *dCBP* inhibitor (Fig. 5c, lanes 3 and 6). In addition, in the Ok107-GAL4 control and H3WT overexpression groups, there were no significance differences in the total courtship time after treatment with ICG-001 (Additional file 6, lanes 1, 4 and lanes 2, 5). However, there were some changes in the H3K23A overexpression group treated with ICG-001 (Additional file 6, lanes 3 and 6). At the same time, we observed a difference in the initial courtship time levels between the H3WT overexpression group and the OK107-GAL4 control group (Additional file 6, lanes 1 and 2) but not in the groups treated with ICG-001 (Additional file 6, lanes 4 and 5). Overall, the similar learning indexes of the H3K23A overexpression mutant and the groups treated with ICG-001 indicated the important role of H3K23ac, which is catalysed by *dCBP.*

Because the *CBP* has a well-established role in neuronal differentiation during development, an immunostaining assay was conducted to study whether the H3K23A mutant plays a role in mushroom body development. Both H3K23A overexpression group and *dCBP* knockdown group did not affect the brain size (Additional file 7a). To our surprise, the mushroom bodies in H3K23A overexpression group was not significantly different from that in the H3WT group, but knockdown of *dCBP* impaired the mushroom bodies (Additional file 7b-c). In addition, treatment with ICG-001 also impaired the development of mushroom bodies like knocking down *dCBP* (Additional file 7d). Because of the mushroom bodies were the key tissue for learning and memory in *Drosophila*, the defect of learning ability might be due to the impaired mushroom bodies in the *dCBP* knockdown flies. To further study the direct role of CBP in courtship learning, we included a courtship learning experiment of the WT flies treated by ICG-001 after eclosion. Because the treating time was much shorter, we increased the dosage of ICG-001 to 10 μM. Similar to the *dCBP* RNAi groups, flies treated with 10 μM ICG-001 after eclosion showed the defect in courtship learning (Additional file 8a), without obvious defect in the structure of the mushroom bodies (Additional file 8b). Above all, these data indicated that *dCBP* plays a role in courtship learning and mushroom body development, although H3K23ac may have a function in regulating learning instead of in mushroom body development.

## Discussion

Cognitive abilities, including learning and memory, are critical for the survival of animals in the environment. In this study, we discovered a previously unknown role for H3K23 acetylation in the regulation of courtship learning in *Drosophila*. We observed a courtship learning defect and impaired neuronal gene activation upon H3K23A mutant overexpression (Fig. 1). Next, our ChIP-qPCR analysis revealed that H3K23ac levels were decreased in these learning-related genes suggesting that the occurrence of H3K23 acetylation was associated with changes in the transcription of neuronal genes related to learning (Fig. 2b and e). Consistent with this hypothesis, knockdown of *dCBP* expression in *Drosophila* also decreased H3K23ac levels and led to a defect in courtship learning ability (Fig. 3e). Moreover, the expression of neuronal genes related to learning was down-regulated in *dCBP* RNAi *Drosophila* due to decreased H3K23ac levels in these genes (Fig. 4a and b). Furthermore, treatment with ICG-001 in the H3K23A mutant overexpression flies did not result in further learning defects, suggesting the important role of *dCBP* related H3K23ac in regulating learning in *Drosophila*. This is, to our knowledge, the first demonstration that H3K23 acetylation, which is regulated by *dCBP*, contributes to courtship learning in *Drosophila*. In summary, our results provide insights into the role of individual chromatin modifications in the learning process.

GFP-tagged histones are a common tool in cellular and developmental biology studies. However, their use may result in some changes in chromatin structure due to their larger size [52]. Overexpression of GFP-tagged histones does not affect neuronal viability but is associated with specific transcriptional and behavioural deficits related to serotonergic dysfunction [52]. In our study, not all learning-related genes showed the same levels of expression and there were differences in the total initial courtship time between the elav-GAL4 control group and the H3WT overexpression group (Fig. 2b), which does not conflict with the previous studies described above. As we are lacking more direct evidence, we cannot explain whether these differences are due to spatial structural differences caused by GFP or the overexpression of histone *H3* itself.

Recent studies have hypothesized that activity-dependent changes in the acetylation of specific lysine residues in neuronal histones can alter the expression of neuroplasticity genes associated with learning and memory formation in a combinatorial manner to encode information related to the history of neuronal activation [13]. In this study, consistent with views of neuroplasticity, decreased H3K23ac levels in neuronal genes impaired their expression and may have led to courtship learning defects in *Drosophila*. This suggested that individual chromatin modifications, such as H3K23ac, might be molecular correlates of learning due to their modulation of the activation of learning-relevant genes.

Given the multiple functions and substrates of HATs and HDACs, genetic evidence alone may be insufficient to definitively demonstrate the involvement of these enzymes in a specific transcriptional process [13]. Here, we identified the causative role of H3K23ac in regulating the expression of learning-related neuronal genes using H3K23A mutants and *dCBP* RNAi flies. Decreased H3K23ac levels in neuronal genes hindered the transcriptional activation of neuronal genes in both H3K23A overexpression and *dCBP* RNAi flies. In addition, the RNA levels of *shakB dnc* and *norpA* were severely reduced after RNAi silencing of *dCBP*. At the same time, both knocking down *dCBP* and inhibiting *dCBP* by ICG-001 impaired the mushroom bodies development, however the mushroom bodies in H3K23A overexpression group was not significantly different from that in the H3WT group. It is possible that *dCBP* may regulate the expression of neuronal genes through other substrates because *dCBP* is a universal acetyltransferase with other histone targets, such as histone H3 lysines 18, 27 and 56, as well as non-histone targets [50, 53, 54]. An immunostaining experiment (Additional file 7) and the abnormal increase in initial courtship time observed in *dCBP* RNAi line (Additional file 6) supported the notion *dCBP* plays roles in various functions besides courtship learning. Our study suggested that H3K23ac may have functions in courtship learning, but not in lone-term memory. Considering the slight change (no significant) in memory index in H3K23A overexpression flies, additional studies are needed to elucidate the mechanisms determining whether H3K23ac is combined with other factors to regulate learning and memory.

## Conclusions

Histone modifications are highly conserved in eukaryotes and have been shown to regulate gene transcription in neuronal processes. Our results presented here suggest an important role for H3K23ac in regulating courtship learning. Overexpression the H3K23A mutant impaired courtship learning. Knocking down *CBP*, a histone acetyltransferase, produced similar phenotypes and attenuated the expression of learning-related genes. In particularly, these two factors did not exert addictive effect on learning regulation in *Drosophila*. This indicating that H3K23ac, which is catalysed by the acetyltransferases *CBP*, regulates the *Drosophila* learning, likely by controlling specific genes.

## Methods

### Fly Stocks

All flies were raised on a standard cornmeal-agar medium at 25°C under 12:12 light/dark conditions unless otherwise stated. The following strains were from the TsingHua Fly Center: UAS-GFP^RNAi^(TH00781.N), UAS-*dCBP*^RNAi^ (THU1718), OK107-GAL4, *Elav*-*GAL4* and *SG-GAL4;* Histone mutants: UAS-H3.3WT-GFP, UAS-H3K23A-GFP, UAS-H3K4A-GFP, UAS-H3K18A-GFP, UAS-H3K37A-GFP, and UAS-H3K122A-GFP. UAS-GFP^RNAi^ and UAS-H3WT-GFP lines were used as controls.

### Courtship Learning and Memory

The methods were based on previous work by us and others [32, 55]. Flies were raised at 25°C and 60–70% humidity in a 12-h/12-h light/dark cycle. When we needed to treat flies with CBP inhibitor, equal volumes of ICG-001 (Selleck) or DMSO were mixed in the food respectively. Flies were collected within 4 hours after eclosion. The males were individually collected in a small food tube (1.5-ml centrifuge tubes containing food). Females were collected in groups in glass tubes with food (approximately 30 animals per tube). Flies were aged for 4-5 days until they reached sexual maturity. On the day before courtship suppression training, a virgin female was paired with a naive male overnight. Then, we collected the mated females.

For the learning assay, males were placed into cells (diameter 15 mm, depth 8 mm) of a wheel chamber via a mouth aspirator. The males adapted to the chamber for 3-5 min. Each trained male was paired with a mated female for 1 hour, and video was recorded during the initial 10 min and last 10 min of 1 hour. After training, males were transferred to a new chamber. For controls (sham training), males were isolated in the chamber for 1 hour.

For the memory training, a male was paired with a mated female in a small food tube (1.5 ml centrifuge tubes with food) for 5 hours. The naïve males were isolated in the tube. After training, males were transferred to a new tube, and their memory was immediately tested. Memory in males was tested by pairing the male with a freeze-kill virgin female, and the test was recorded for 10 min.

For each 10 min recording, we calculated a courtship index (CI) for each male: CI_initial_, CI_final_, CI_test_ and CI_sham_. The CI is the percent of time males exhibited courtship behaviour (orientation, wing vibration, licking, and attempted copulation) during the 10 min recording. If copulation occurred during training, or the male scored CI_initial_<0.1, the data were discarded. The learning index (LI) was also calculated. The LI is the ratio of the courtship level during the final 10 min of training (CI_final_) to the initial 10 min. The memory index was calculated by dividing CI_test_ by the mean of the sham control courtship levels, CI_sham_. An LI or MI score ≥1 indicates no learning or memory.

**LI=**CI_final_/ CI_initial_ **MI=** CI_test_/ mean CI_sha**m**_

### Immunostaining of the Salivary Glands and Mushroom Bodies

We performed immunofluorescence as previously described[32]. The salivary glands were dissected from third-instar larva and the brains were dissected from adult heads, respectively. Then fixed the samples in fixation solution (3.7% formaldehyde and 1% Triton X-100 in PBS) for 6 min. Next, the fixation solution was discarded, and the glands were incubated in dissociation solution (3.7% formaldehyde and 50% acetic acid in ddH_2_O) for 10 min. The samples were washed in PBS three times. Then, the samples were incubated with the following antibodies at 4°C overnight: H3K23ac (1:50; ab46982; Abcam); *Fas2* (1:100; DSHB). After the primary antibody incubation, the salivary glands were incubated with secondary antibody (1:200; 488) for 1 hour and stained with DAPI (1:1000) for 10 min. Finally, the samples were observed with a fluorescence microscope.

### Western Blotting Assays

In total, 120 pairs of salivary glands or brains from third-instar larva were lysed in 200 μl of 4% SDS. The lysates were incubated in 50 μl of 5× SDS loading buffer and boiled for 10 min followed by centrifugation at 14,000 × *g* for 3 min at 4°C to collect the supernatants. Protein concentration was measured with a BCA protein assay kit (cwbiotech), and equal amounts of protein were loaded to perform western blotting. The following antibodies were used: H3K23ac (1:1000; ab46982; Abcam), H3 (1:1000; ab1791; Abcam), H3K23me1 (1:1000; 39387; Active motive).

### RNA Purification and RT-qPCR

A total of 50 larval heads for one sample were homogenized with TRIzol reagent (Takara) according to the manufacturers’ instructions. 2 μg of total RNA was utilized for cDNA synthesis with an OligodT primer (Takara) and M-MLV reverse transcriptase (Promega). The generated cDNA was utilized for RT-qPCR. All PCR reactions were performed in an ABI 7500 system. The following primers were used:

*para* F- AGGACTTCTGGGAGGATCTGT

*para* R- AGCTTCTTCCGCTTCACGTA

*shakB* F- GCAAAATATGGAGTTTTTACGCGG

*shakB* R- CATCTTCGGGAATGTCTCTTGTATG

*Hk* F- CAATCGTAATGCCGCTCTGTC

*Hk* R- ACTGGCTCATGTTTCAGCGA

*Sh* F-GCACAAAAATCGAGGAAGACGA

*Sh* R-GGACATGCGAGGAACCTGA

*cngl* F- ATATACGATGGAAGCGCACGA

*cngl* R-GAGATACTGGTGCCCGTGG

*SK* F-TCGCCTGGGAAAGAGGAAAG

*SK* R-CGAGTAGAACGATGCCTTTGTG

*dnc* F-TACATCGTCCACCCGCTATG

*dnc* R-GGGTTACTTGAAAGCGTATCCTG

*norpA* F-CGTCATGCCAGGGACATCAA

*norpA* R-GCCGACGATCCTTCTCGTT

*gfA* F-GTGGACCTACGATGGGTTTCA

*gfA* R-TACCGGGCACACCTAACAAG

*eag* F- ACACGAAGGGAGTTTTAGGTCTC

*eag* R- GGAGTATGTGTGGTGGCGT

*dCBP* F- TCGCAAACCCAAATCAAAAAGGA

*dCBP* R-CAGTTATCGCACACGAAGCC

*rp49* F-AAGCTGTCGCACAAATGGC

*rp49* R-CTTCTTGAATCCGGTGGGCA

*act5c* F-AGTCGGTTTATTCCAGTCATTCCTT

*act5c* R- AGAGCAGCAACTTCTTCGTCA

*αTub84B* F- CTGTGAATTTTCCTTGTCGCGT

*αTub84B* R- CCAGCAGGCGTTTCCAATC

*βTub56D* F-GCGCCAAGTTCTGGGAGAT

*βTub56D* R-CACCGGACGCCTCATTGTA

*gapdh1* F- CCCAATGTCTCCGTTGTGGA

*gapdh1* R-CATCGGTGTAGCCCAGGATT

rp49 was considered a reference gene.

### ChIP analysis

The ChIP assay was performed as previously described [32]. dCBP RNAi virgin females were crossed with *Elav*-*GAL4* males, and the GFP RNAi strain was crossed with the same GAL4 as a control. For one chromatin immunoprecipitation, approximately 400 brain-disc complexes were dissected from third-instar larvae and stored at -80°C. Tissue fixation was conducted in PBS with 1% formaldehyde for 20 min at room temperature, and the process was stopped by the addition of 125 mM glycine for 5 min. After two washes with PBS, the tissues were lysed in RIPA buffer (50 mM Tris-HCl pH 8.0, 150 mM NaCl, 2 mM EDTA pH 8.0, 1% NP-40, 0.5% sodium deoxycholate, 0.1% SDS) and were sonicated for 10 min (power output: 25%, 2 seconds on and 4 seconds off). This sonication method yields genomic DNA fragments of 150-500 bp. The soluble chromatin was collected by centrifugation at 4°C for 10 min at 12000 rpm. 10 μl aliquots of supernatants were used to estimate chromatin concentrations after reverse cross-linking (input DNA). A total of 15 μg of genomic DNA was incubated with 10 μl of antibody at 4°C overnight. The antibody-chromatin complexes were captured with the addition of 30 μl of Protein A/G-Agarose beads suspension (Santa Cruz Biotechnology). The beads were washed three times with RIPA buffer and once with elution buffer (50 mM NaHCO_3_, 1% SDS). After reversal crossing-linking, input DNA and immunoprecipitation DNA were purified via phenol-chloroform extraction. RT-qPCR was performed to measure the enrichment of H3k23ac on target genes.

The following primers were used:

*SK* F-TGATGGACACGCAGTTGAC

*SK* R-TTGGTGGGTTCTTACACGG

*para* F-CTGCCACAAAGACCCATAC

*para* R-AAATCGAGCCAACACCAC

*eag* F-GTTACCACCATCATCCAGC

*eag* R- CTCGGTATCCAGACCCTTG

*shakB* F-TGAGACGGCAGACAGAGC

*shakB* R-TTCTTCCTGGGTCCTTCAGC

*Sh* F-TACTTGTTGCTGGCGGGTC

*Sh* R-AATGGGTTCAAAGTGTTGCG

*cngl* F-GTGGTCACTCCCTATCCG

*cngl* R-CATGTCCGTCTCACTAAATTCC

*norpA* F-TCAACAGCTCGCATAATACC

*norpA* R-CTCGTCCTCACCCTTTCC

*gfA* F-AGAAGCCATCACGCAGTC

*gfA* R-AAGTCCCAGGCCATAGACC

*Hk* F- ACGGGCGGTCATCTTGATTT

*Hk* R- ACCCGCTTTTGCCCAGATT

*dnc* F- GACAGTATGCACGCTCCC

*dnc* R-CCAGCTCATTCTCCCTGG

intergenic region F-GCTGATGCTTCCTGAAATCC

intergenic region R-GTTTGGTGTGCTCGTCCTTT

### Calcium fluorescence imaging

Calcium imaging was performed following previously described protocols [56, 57]. Larvae brains were dissected in HL3 solution, and were incubated with fura-red (60um) for 20 min at 37°C. After washing brain with HL3 solution, brains were imaged with the fluorescence microscope. Record baseline for 2 min, and add KCL (100mM). The time record fluorescence is a total of 900 s. For each region of interest (brain lobe), the baseline rate (R_0_) was calculated by the fluorescence intensity of fura-red dividing by that of GFP preceding the stimulus. △R/R_0_ was calculated by relative change in fluorescence intensity normalized to the baseline fluorescence. This is a negative deflection when the intracellular Ca^2+^ concentration increases using fura-red as the Ca^2+^ indicator dye. So, we multiplied the ΔR/R_0_ values with -1 to obtain positive signals to display a rise in Ca^2+^.

## Additional files

Additional file 1: Histone mutants are incorporated into the chromatin. Salivary gland immunostaining was performed in histone H3 mutant flies with anti-GFP. The overexpression of Histone mutants was driven by SG-GAL4. Scale bars: 50 μm. (DOCX 440 kb)

Additional file 2: Training for 5 hours made the learning index of H3K23A overexpression group at the similar level to the H3WT group. A courtship learning experiment was conducted to value the learning levels after training 5 hours. Each male fly was allowed to train for 5 hours as the method described. The learning index of courtship was the time spent during the final 10 min vs. the initial 10 min. Unpaired t-test was used for statistics. Error bars represent the standard error of the mean; the number of samples was indicated in the bar. n.s., not significant. p=0.1379. (DOCX 50 kb)

Additional file 3: The total initial courtship time do not affect courtship learning indexes. (a-e) The data of total courtship time were from the courtship learning and memory experiments. Flies overexpressing H3K23A, H3K4A, H3K18A and H3K122A were compared to H3WT overexpression group, respectively. Except for the group of overexpressing the H3K37A, there were no significant change in the total courtship time among other groups of flies. (f-k) The learning index data were divided into two subgroups by the median. Then the learning indexes are compared to another subgroups. These analyses were used to study whether lower CI has an impact on the learning index. Unpaired Two-tailed Student’s t-test was used. Error bars represent the standard error of the mean; the number of samples was indicated in the bar. n.s., not significant. *p<0.05, **p<0.01. (DOCX 240 kb)

Additional file 4: Overexpression of the H3K23A mutants affected the gene expression and Calcium signaling. (a) Four housekeeping genes were examined by RT-qPCR. The samples were from larval brains. The expression levels were normalized to the levels of rp49. No significant difference was observed in this experiment. (b) Calcium signalling was impaired in H3K23A mutants during KCl stimulus. The baseline rate (R0) was calculated by the fluorescence intensity of fura-red dividing by that of GFP preceding the stimulus. △R/R0 was calculated by relative change in fluorescence intensity normalized to the baseline fluorescence. n= 6 for each group. (DOCX 187 kb)

Additional file 5: The total initial courtship time in the GFP RNAi flies and the *dCBP* RNAi flies. (a) The data of total courtship time were from the flies with Ok107-GAL4 derived RNAi in courtship learning experiments. (b-c) The learning index data of either GFP RNAi groups or *dCBP* RNAi groups were divided into two subgroups by the median. Then the learning indexes are compared to another subgroups. Unpaired Two-tailed Student’s t-test was used. Error bars represent the standard error of the mean; the number of samples was indicated in the bar. n.s., not significant. ***p<0.001. (DOCX 149 kb)

Additional file 6: The total initial courtship time of the groups with or without treatment of ICG-001. The data of total courtship time were from the courtship learning experiments. Unpaired t-test was used. Error bars represent the standard error of the mean; the number of samples was indicated in the bar. n.s., not significant. *p<0.05, ##p<0.01 (elav-GAL4 group compared to H3WT group). (DOCX 71 kb)

Additional file 7: The structure of mushroom body in H3K23A overexpression and *dCBP* RNAi flies. (a) Immunostaining of H3K23ac in the adult brains were conducted for observing the morphology of brains. (b-d) The mushroom bodies were stained by anti-fas2 antibodies. (b) It seems to be no significant change in mushroom bodies in the group of overexpressing the H3K23A mutant. (c) The defects of mushroom bodies were observed in the flies knockdown *dCBP*. (d) The same experiment was used for detecting whether ICG-001 impaired the development of mushroom bodies. n=3. (DOCX 1213 kb)

Additional file 8: Treatment with ICG-001 in adult flies affected the courtship learning. (a) A courtship learning experiment was included to test whether treatment with ICG-001 in adult flies led to impair the learning ability. The flies were treated with 10μM of ICG-001 for 4 to 5 days after eclosion. The learning index of courtship was the time spent during the final 10 min vs. the initial 10 min. Unpaired t-test was used for statistics. Error bars represent the standard error of the mean; the number of samples was indicated in the bar. *, p<0.05. (b) The mushroom bodies were stained by anti-fas2 antibodies. Scale bars: 50 μm. n=5. (DOCX 443 kb)

Additional file 9: Representative movies of H3WT, H3K23A, GFP RNAi and *dCBP* RNAi fly courtship learning. Videos were recorded during the initial and final 10 min of the 60 min training session. Video 1: H3WT flies exhibited courtship suppression after training. Video 2: H3K23A flies exhibited a reduction of courtship suppression and a high courtship index after training. Video 3: GFP RNAi flies exhibited normal courtship suppression after training. Video 4: *dCBP* RNAi flies exhibited less courtship suppression after training. (ZIP 25330 kb)

## Abbreviations

5-HT: 5-hydroxytryptamine
C/EBP: CAAT box enhancer binding protein
CBP: CREB-binding protein
GNATs: Gcn5–related N-Acetyltransferases
