# Supplementary material for: Histone acetyltransferase CBP-related H3K23 acetylation contributes to courtship learning in Drosophila
Source: BMC Dev Biol. 2018 Nov 20;18:20. doi: 10.1186/s12861-018-0179-z (PMC6247617; doi:10.1186/s12861-018-0179-z)
Supplement: Supplementary file 1 — Histone mutants are incorporated into the chromatin. Salivary gland immunostaining was performed in histone H3 mutant flies with anti-GFP. The overexpression of Histone mutants was driven by SG-GAL4. Scale bars: 50 μm. (DOCX 440 kb) [file 12861_2018_179_MOESM1_ESM.docx]

**
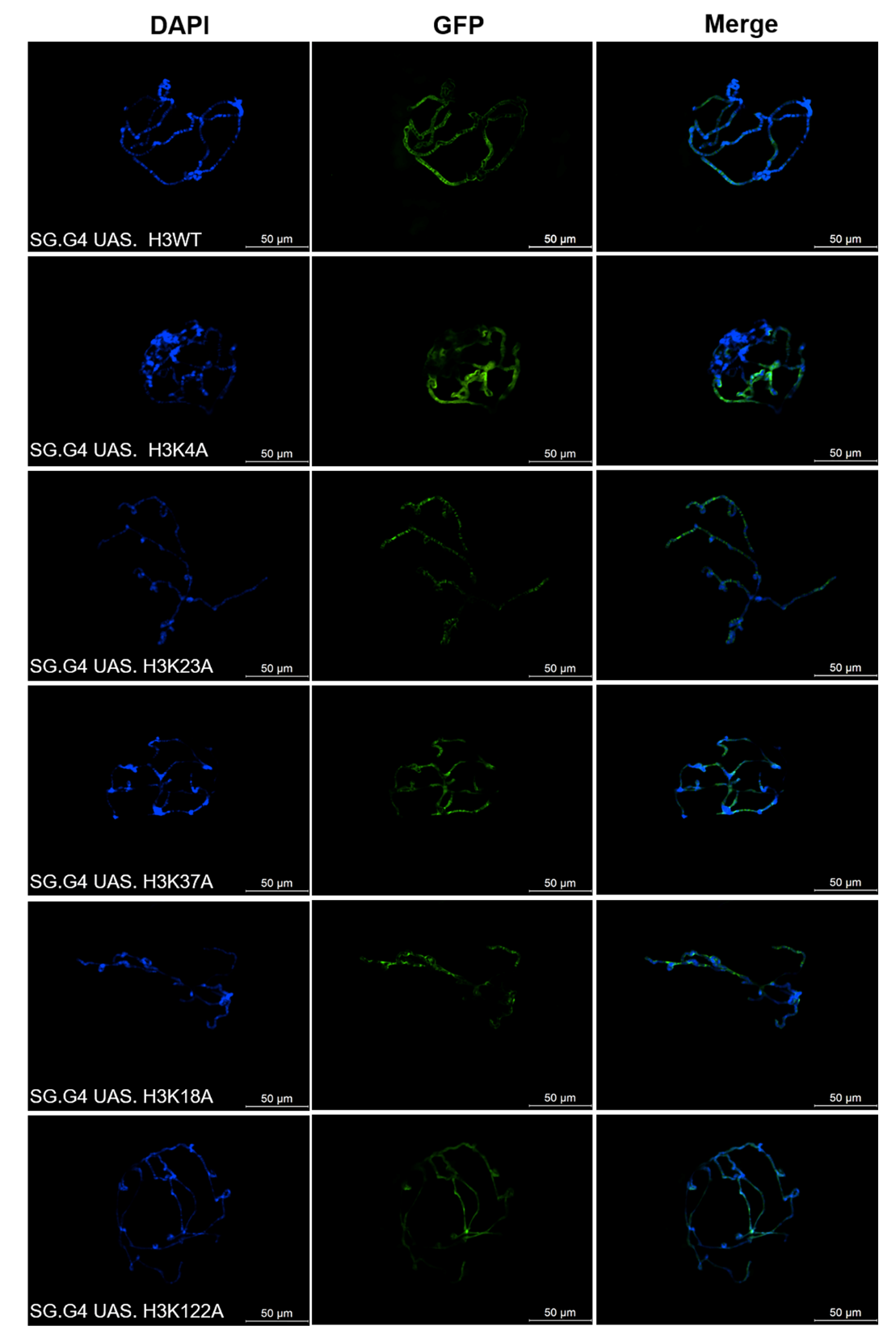
**

**Additional file 1. Histone mutants are incorporated into the chromatin.** Salivary gland immunostaining was performed in histone H3 mutant flies with anti-GFP**.** The overexpression of Histone mutants was driven by SG-GAL4. Scale bars: 50 μm.
