# Supplementary material for: Histone acetyltransferase CBP-related H3K23 acetylation contributes to courtship learning in Drosophila
Source: BMC Dev Biol. 2018 Nov 20;18:20. doi: 10.1186/s12861-018-0179-z (PMC6247617; doi:10.1186/s12861-018-0179-z)
Supplement: Supplementary file 2 — Training for 5 hours made the learning index of H3K23A overexpression group at the similar level to the H3WT group. A courtship learning experiment was conducted to value the learning levels after training 5 hours. Each male fly was allowed to train for 5 hours as the method described. The learning index of courtship was the time spent during the final 10 min vs. the initial 10 min. Unpaired t-test was used for statistics. Error bars represent the standard error of the mean; the number of samples was indicated in the bar. n.s., not significant. p=0.1379. (DOCX 50 kb) [file 12861_2018_179_MOESM2_ESM.docx]

**
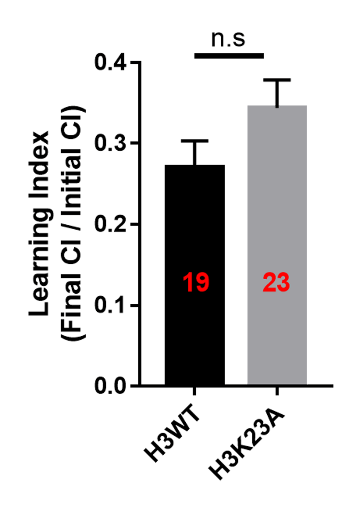
**

**Additional file 2. Training for 5 hours made the learning index of H3K23A overexpression group at the similar level to the H3WT group.** A courtship learning experiment was conducted to value the learning levels after training 5 hours. Each male fly was allowed to train for 5 hours as the method described. The learning index of courtship was the time spent during the final 10 min vs. the initial 10 min. Unpaired t-test was used for statistics. Error bars represent the standard error of the mean; the number of samples was indicated in the bar. n.s., not significant. p=0.1379.
