# Supplementary material for: Histone acetyltransferase CBP-related H3K23 acetylation contributes to courtship learning in Drosophila
Source: BMC Dev Biol. 2018 Nov 20;18:20. doi: 10.1186/s12861-018-0179-z (PMC6247617; doi:10.1186/s12861-018-0179-z)
Supplement: Supplementary file 3 — The total initial courtship time do not affect courtship learning indexes. (a-e) The data of total courtship time were from the courtship learning and memory experiments. Flies overexpressing H3K23A, H3K4A, H3K18A and H3K122A were compared to H3WT overexpression group, respectively. Except for the group of overexpressing the H3K37A, there were no significant change in the total courtship time among other groups of flies. (f-k) The learning index data were divided into two subgroups by the median. Then the learning indexes are compared to another subgroups. These analyses were used to study whether lower CI has an impact on the learning index. Unpaired Two-tailed Student’s t-test was used. Error bars represent the standard error of the mean; the number of samples was indicated in the bar. n.s., not significant. *p<0.05, **p<0.01. (DOCX 240 kb) [file 12861_2018_179_MOESM3_ESM.docx]

**
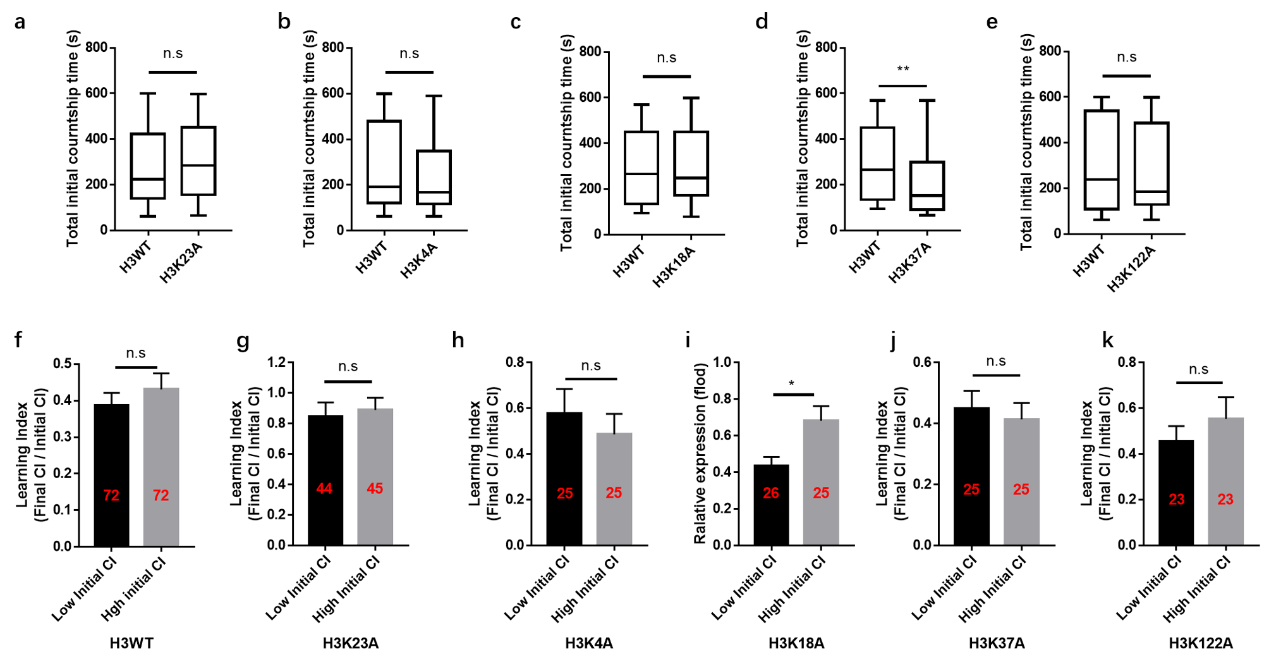
**

**Additional file 3. The total initial courtship time do not affect courtship learning indexes.**  (a-e) The data of total courtship time were from the courtship learning and memory experiments. Flies overexpressing H3K23A, H3K4A, H3K18A and H3K122A were compared to H3WT overexpression group, respectively. Except for the group of overexpressing the H3K37A, there were no significant change in the total courtship time among other groups of flies. (f-k) The learning index data were divided into two subgroups by the median. Then the learning indexes are compared to another subgroups. These analyses were used to study whether lower CI has an impact on the learning index. Unpaired Two-tailed Student’s t-test was used. Error bars represent the standard error of the mean; the number of samples was indicated in the bar. n.s., not significant. *p<0.05, **p<0.01.
