# Supplementary material for: Histone acetyltransferase CBP-related H3K23 acetylation contributes to courtship learning in Drosophila
Source: BMC Dev Biol. 2018 Nov 20;18:20. doi: 10.1186/s12861-018-0179-z (PMC6247617; doi:10.1186/s12861-018-0179-z)
Supplement: Supplementary file 4 — Overexpression of the H3K23A mutants affected the gene expression and Calcium signaling. (a) Four housekeeping genes were examined by RT-qPCR. The samples were from larval brains. The expression levels were normalized to the levels of rp49. No significant difference was observed in this experiment. (b) Calcium signalling was impaired in H3K23A mutants during KCl stimulus. The baseline rate (R0) was calculated by the fluorescence intensity of fura-red dividing by that of GFP preceding the stimulus. △R/R0 was calculated by relative change in fluorescence intensity normalized to the baseline fluorescence. n= 6 for each group. (DOCX 187 kb) [file 12861_2018_179_MOESM4_ESM.docx]

**
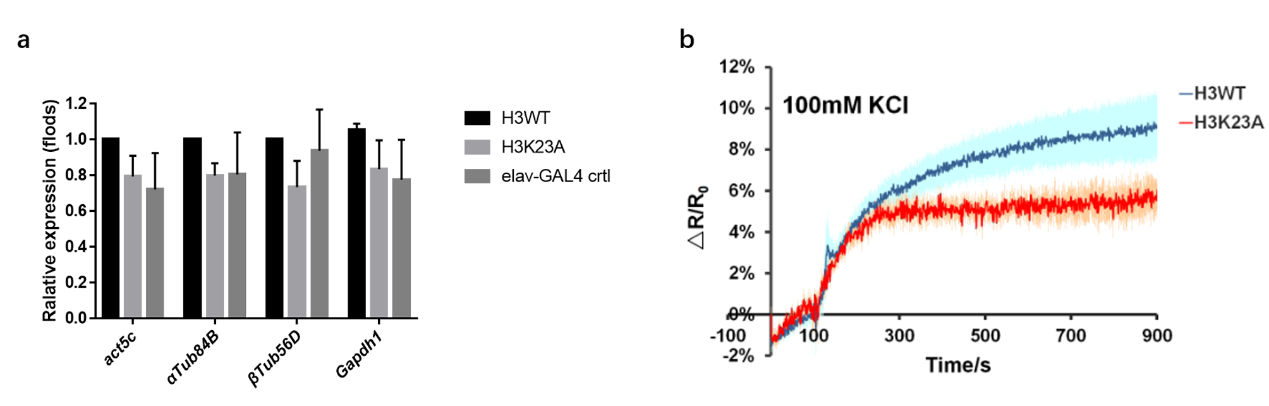
**

**Additional file 4. Overexpression of the H3K23A mutants affected the gene expression and Calcium signaling.** (a) Four housekeeping genes were examined by RT-qPCR. The samples were from larval brains. The expression levels were normalized to the levels of rp49. No significant difference was observed in this experiment. (b) Calcium signalling was impaired in H3K23A mutants during KCl stimulus. The baseline rate (R0) was calculated by the fluorescence intensity of fura-red dividing by that of GFP preceding the stimulus. △R/R0 was calculated by relative change in fluorescence intensity normalized to the baseline fluorescence. n= 6 for each group.
