# Supplementary material for: Histone acetyltransferase CBP-related H3K23 acetylation contributes to courtship learning in Drosophila
Source: BMC Dev Biol. 2018 Nov 20;18:20. doi: 10.1186/s12861-018-0179-z (PMC6247617; doi:10.1186/s12861-018-0179-z)
Supplement: Supplementary file 5 — The total initial courtship time in the GFP RNAi flies and the dCBP RNAi flies. (a) The data of total courtship time were from the flies with Ok107-GAL4 derived RNAi in courtship learning experiments. (b-c) The learning index data of either GFP RNAi groups or dCBP RNAi groups were divided into two subgroups by the median. Then the learning indexes are compared to another subgroups. Unpaired Two-tailed Student’s t-test was used. Error bars represent the standard error of the mean; the number of samples was indicated in the bar. n.s., not significant. ***p<0.001. (DOCX 149 kb) [file 12861_2018_179_MOESM5_ESM.docx]

**
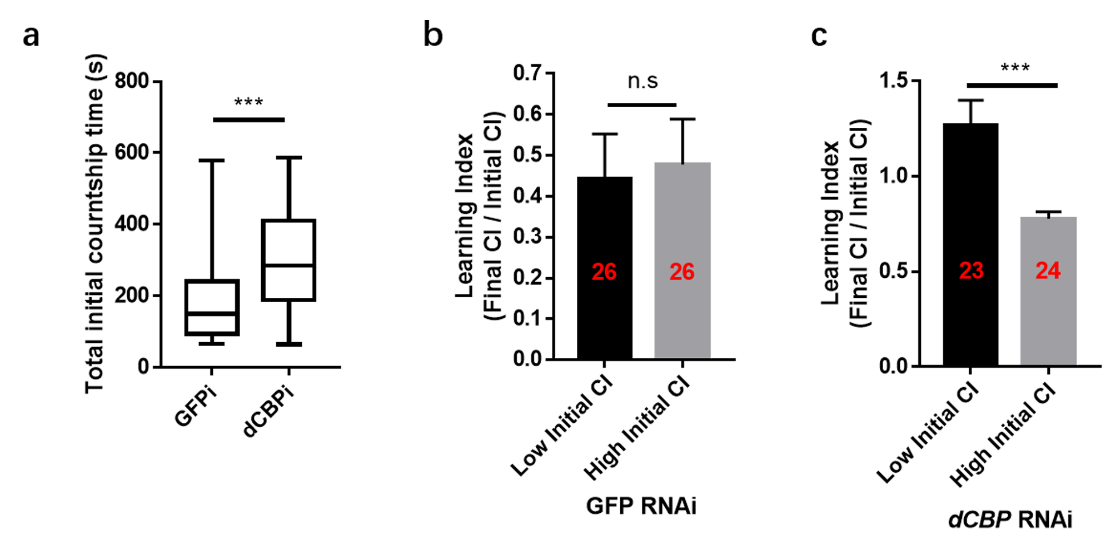
**

**Additional file 5. The total initial courtship time in the GFP RNAi flies and the *dCBP* RNAi flies.**  (a) The data of total courtship time were from the flies with Ok107-GAL4 derived RNAi in courtship learning experiments. (b-c) The learning index data of either GFP RNAi groups or *dCBP* RNAi groups were divided into two subgroups by the median. Then the learning indexes are compared to another subgroups. Unpaired Two-tailed Student’s t-test was used. Error bars represent the standard error of the mean; the number of samples was indicated in the bar. n.s., not significant. ***p<0.001.
