# Supplementary material for: Histone acetyltransferase CBP-related H3K23 acetylation contributes to courtship learning in Drosophila
Source: BMC Dev Biol. 2018 Nov 20;18:20. doi: 10.1186/s12861-018-0179-z (PMC6247617; doi:10.1186/s12861-018-0179-z)
Supplement: Supplementary file 6 — The total initial courtship time of the groups with or without treatment of ICG-001. The data of total courtship time were from the courtship learning experiments. Unpaired t-test was used. Error bars represent the standard error of the mean; the number of samples was indicated in the bar. n.s., not significant. *p<0.05, ##p<0.01 (elav-GAL4 group compared to H3WT group). (DOCX 71 kb) [file 12861_2018_179_MOESM6_ESM.docx]

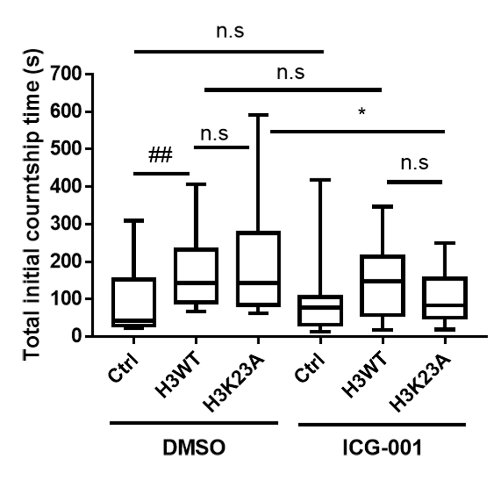


**Additional file 6. The total initial courtship time of the groups with or without treatment of ICG-001.** The data of total courtship time were from the courtship learning experiments. Unpaired t-test was used. Error bars represent the standard error of the mean; the number of samples was indicated in the bar. n.s., not significant. *p<0.05, ##p<0.01 (elav-GAL4 group compared to H3WT group).
