# Supplementary material for: Histone acetyltransferase CBP-related H3K23 acetylation contributes to courtship learning in Drosophila
Source: BMC Dev Biol. 2018 Nov 20;18:20. doi: 10.1186/s12861-018-0179-z (PMC6247617; doi:10.1186/s12861-018-0179-z)
Supplement: Supplementary file 7 — The structure of mushroom body in H3K23A overexpression and dCBP RNAi flies. (a) Immunostaining of H3K23ac in the adult brains were conducted for observing the morphology of brains. (b-d) The mushroom bodies were stained by anti-fas2 antibodies. (b) It seems to be no significant change in mushroom bodies in the group of overexpressing the H3K23A mutant. (c) The defects of mushroom bodies were observed in the flies knockdown dCBP. (d) The same experiment was used for detecting whether ICG-001 impaired the development of mushroom bodies. n=3. (DOCX 1213 kb) [file 12861_2018_179_MOESM7_ESM.docx]

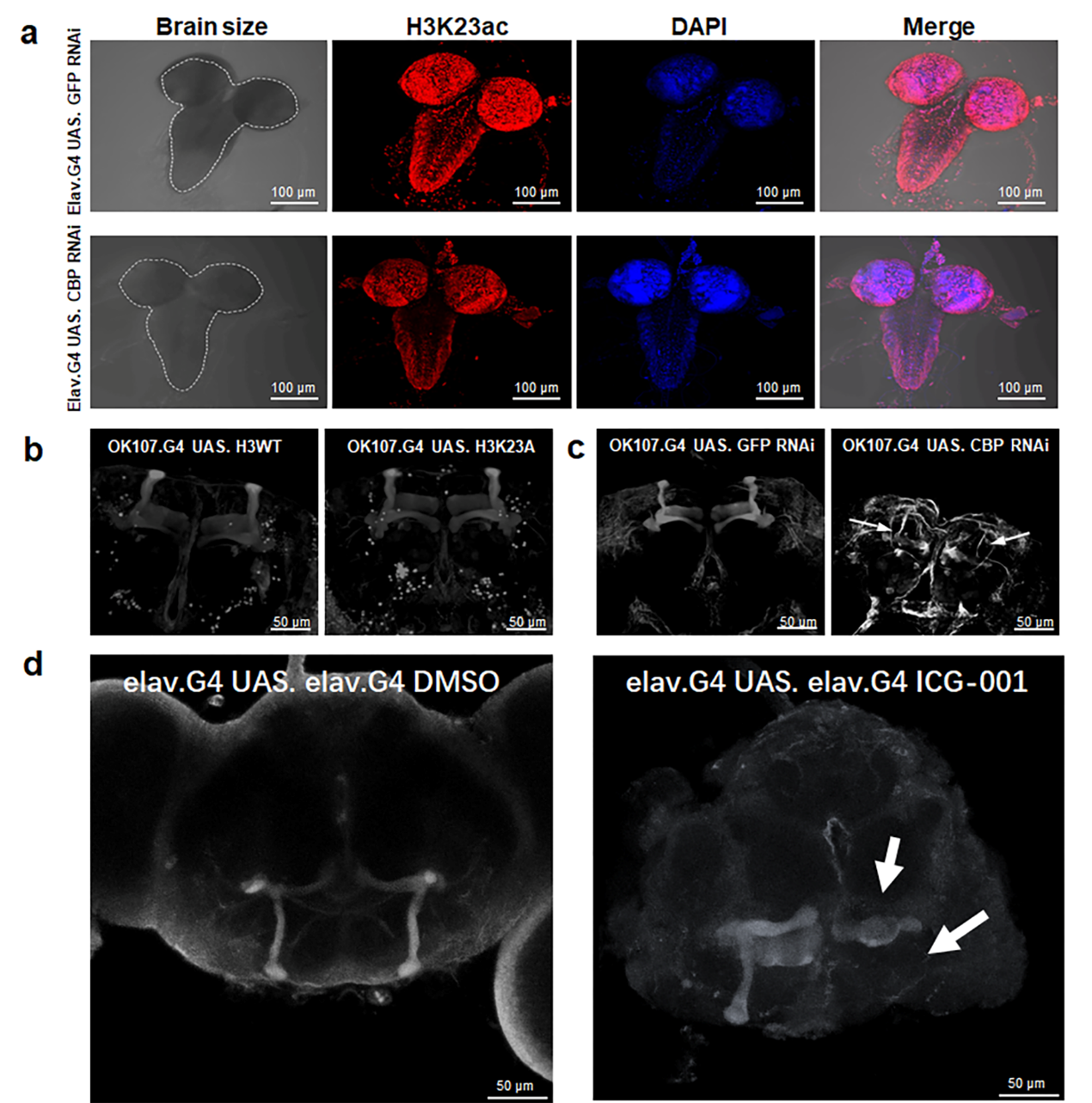


**Additional file 7. The structure of mushroom body in H3K23A overexpression and *dCBP* RNAi flies.** (a) Immunostaining of H3K23ac in the adult brains were conducted for observing the morphology of brains. (b-d) The mushroom bodies were stained by anti-fas2 antibodies. (b) It seems to be no significant change in mushroom bodies in the group of overexpressing the H3K23A mutant. (c) The defects of mushroom bodies were observed in the flies knockdown *dCBP.* (d) The same experiment was used for detecting whether ICG-001 impaired the development of mushroom bodies. n=3
