# Supplementary material for: Histone acetyltransferase CBP-related H3K23 acetylation contributes to courtship learning in Drosophila
Source: BMC Dev Biol. 2018 Nov 20;18:20. doi: 10.1186/s12861-018-0179-z (PMC6247617; doi:10.1186/s12861-018-0179-z)
Supplement: Supplementary file 8 — Treatment with ICG-001 in adult flies affected the courtship learning. (a) A courtship learning experiment was included to test whether treatment with ICG-001 in adult flies led to impair the learning ability. The flies were treated with 10μM of ICG-001 for 4 to 5 days after eclosion. The learning index of courtship was the time spent during the final 10 min vs. the initial 10 min. Unpaired t-test was used for statistics. Error bars represent the standard error of the mean; the number of samples was indicated in the bar. *, p<0.05. (b) The mushroom bodies were stained by anti-fas2 antibodies. Scale bars: 50 μm. n=5. (DOCX 443 kb) [file 12861_2018_179_MOESM8_ESM.docx]

**
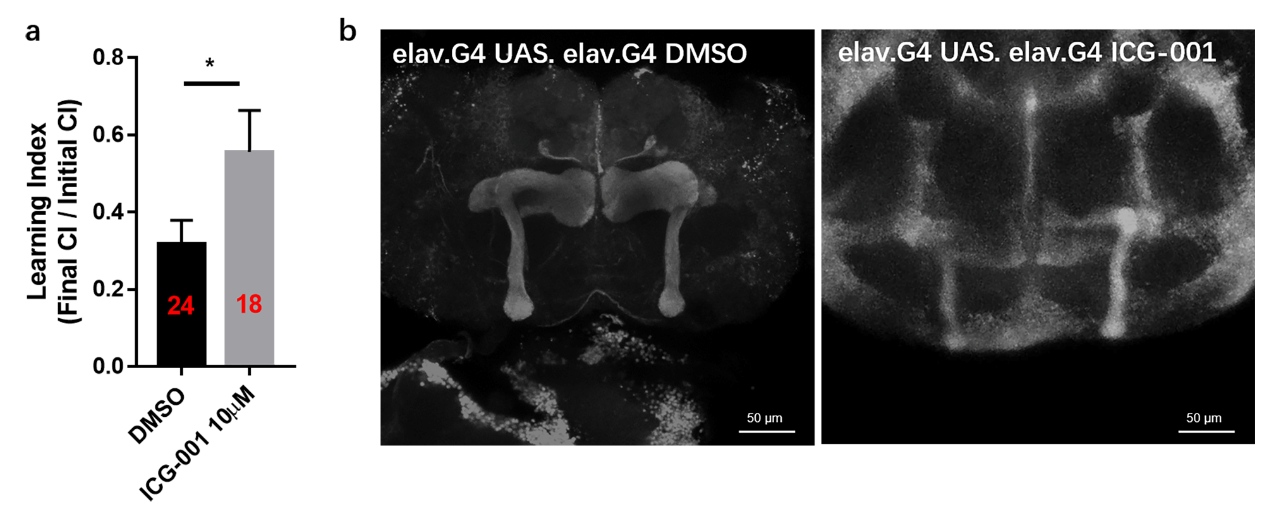
**

**Additional file 8. Treatment with ICG-001 in adult flies affected the courtship learning.** (a) A courtship learning experiment was included to test whether treatment with ICG-001 in adult flies led to impaired the learning ability. The flies were treated with 10μM of ICG-001 for 4 to 5 days after eclosion. The learning index of courtship was the time spent during the final 10 min vs. the initial 10 min. Unpaired t-test was used for statistics. Error bars represent the standard error of the mean; the number of samples is indicated in the bar. *, p<0.05. (b) The mushroom bodies were stained by anti-fas2 antibodies. Scale bars: 50 μm. n=5
